# Rapid and Efficient Polymer/Contaminant Removal from Single-Layer Graphene via Aqueous Sodium Nitrite Rinsing for Enhanced Electronic Applications

**DOI:** 10.3390/polym17050689

**Published:** 2025-03-04

**Authors:** Kimin Lee, Juneyoung Kil, JaeWoo Park, Sui Yang, Byoungchoo Park

**Affiliations:** 1 Department of Electrical and Biological Physics, Kwangwoon University, Wolgye-Dong, Seoul 01897, Republic of Korea; dlrlals1123@naver.com (K.L.); jun097@naver.com (J.K.); 2 Materials Science and Engineering, School for Engineering of Matter Transport and Energy, Arizona State University, Tempe, AZ 85287, USA; jpark441@asu.edu (J.P.); suiyang@asu.edu (S.Y.)

**Keywords:** CVD, single-layer graphene, sodium nitrite rinsing, surface residues, PMMA polymer, Cl^−^ ions, doping

## Abstract

The removal of surface residues from single-layer graphene (SLG), including poly(methyl methacrylate) (PMMA) polymers and Cl^−^ ions, during the transfer process remains a significant challenge with regard to preserving the intrinsic properties of SLG, with the process often leading to unintended doping and reduced electronic performance capabilities. This study presents a rapid and efficient surface treatment method that relies on an aqueous sodium nitrite (NaNO_2_) solution to remove such contaminants effectively. The NaNO_2_ solution rinse leverages reactive nitric oxide (NO) species to neutralize ionic contaminants (e.g., Cl^−^) and partially oxidize polymer residues in less than 10 min, thereby facilitating a more thorough final cleaning while preserving the intrinsic properties of graphene. Characterization techniques, including atomic force microscopy (AFM), Kelvin probe force microscopy (KPFM), and X-ray photoelectron spectroscopy (XPS), demonstrated substantial reductions in the levels of surface residues. The treatment restored the work function of the SLG to approximately 4.79 eV, close to that of pristine graphene (~4.5–4.8 eV), compared to the value of nearly 5.09 eV for conventional SLG samples treated with deionized (DI) water. Raman spectroscopy confirmed the reduced doping effects and improved structural integrity of the rinsed SLG. This effective rinsing process enhances the reproducibility and performance of SLG, enabling its integration into advanced electronic devices such as organic light-emitting diodes (OLEDs), photovoltaic (PV) cells, and transistors. Furthermore, the technique is broadly applicable to other two-dimensional (2D) materials, paving the way for next-generation (opto)electronic technologies.

## 1. Introduction

Graphene, a two-dimensional (2D) carbon nanomaterial arranged in a hexagonal lattice, has attracted considerable attention due to its exceptional electronic, mechanical, and optical properties [1,2,3,4,5,6,7]. With remarkable electron mobility and superior charge transport capabilities, graphene is poised to revolutionize next-generation electronics, photonics, and biosensors [8,9,10,11]. The increasing demand for transparent, flexible, and conductive materials has further driven comparative studies of graphene alongside alternatives such as metallic nanowires [12,13,14,15] and carbon nanotubes [16,17]. Among these, single-layer graphene (SLG) stands out for its excellent optical transmittance and electrical conductivity, positioning it as a prime candidate to replace conventional brittle indium tin oxide (ITO) in optoelectronic devices. Potential applications include organic light-emitting diodes (OLEDs), photovoltaic (PV) cells, and transistors [18,19,20,21,22].

To realize practical applications, large-area SLG specimens are typically synthesized by chemical vapor deposition (CVD) on metal substrates such as copper (Cu) and subsequently transferred to target substrates such as SiO_2_-covered silicon (Si) or hexagonal boron nitride (BN) using polymeric scaffolds such as poly(methyl methacrylate) (PMMA) [21,22,23,24,25,26]. While this transfer process is essential for device fabrication, it inevitably results in surface contamination with polymer residues, etchant salts, and other chemicals used during the transfer. Such residues can deteriorate the intrinsic properties of graphene by introducing unintended doping or creating scattering sites, thereby degrading its electrical performance [24,25,26,27,28,29,30,31,32]. For instance, the adsorption of chemicals such as PMMA, FeCl_3_, and Fe(NO_3_)_3_ during the transfer process results in doping contamination, which is challenging to eliminate through conventional rinsing methods using deionized (DI) water or organic solvents, even with extended rinsing times [24,25,26,27,28,31,32,33,34,35].

To address these challenges, several advanced techniques for the cleaning of graphene surfaces have been investigated [25,26]. High-temperature annealing in an inert atmosphere [36,37,38], electrostatic force cleaning [39,40], and plasma treatments [36,41] have shown effectiveness in removing residues but have significant limitations. High-temperature methods are unsuitable for temperature-sensitive substrates, while plasma and electrostatic treatments are often associated with difficulties in controlling the particle energy, risking damage to the delicate graphene lattice [36,39]. Recently, electrochemical treatments have emerged as promising alternatives; however, methods involving faradaic or non-faradaic processes can cause unwanted damage and can lack uniformity over the graphene area [39,42,43].

In this work, we present a two-step cleaning strategy comprising a rinse with an aqueous sodium nitrite (NaNO_2_) solution followed by washes with organic solvents. We show that NaNO_2_ effectively targets Cl^−^ doping and weakens PMMA adhesion, thereby ensuring a significantly cleaner and less doped graphene surface compared to conventional DI water treatments. In particular, NaNO_2_ rinsing can address both ionic contaminants (notably Cl^−^) and partially loosen residual polymers on graphene, enabling a more thorough solvent-based removal of PMMA. This method is integrated into the graphene transfer process, facilitating the desorption of PMMA and Cl^−^ residues within a short processing time of approximately 10 min without the need for post-thermal annealing or an electrochemical system. Reactive nitric oxide (NO) species generated in the NaNO_2_ solution primarily target ionic contaminants (e.g., Cl^−^) and partially oxidize polymeric residues, thereby weakening their adhesion to graphene through a synergistic combination of mild chemical oxidation and physical desorption mechanisms. Atomic force microscopy (AFM), Kelvin probe force microscopy (KPFM), X-ray photoelectron spectroscopy (XPS), and Raman spectroscopy analyses confirm significant reductions in the surface roughness and the effective removal of contaminants, thereby restoring the intrinsic electrical properties of SLG.

The NaNO_2_ aqueous solution rinsing approach not only ensures the integrity of the graphene lattice but also provides a foundation for precise *n*- or *p*-doping, which is essential for the reliable and reproducible fabrication of high-performance graphene-based (opto)electronic devices. By overcoming the critical challenges of residue-induced doping and scattering, our method paves the way for the development of advanced electronic devices, including OLEDs, PV cells, and transistors, with improved performance and stability. Furthermore, the principles underlying this cleaning technique can be extended to other 2D materials, broadening its impact across materials science and device engineering.

## 2. Materials and Methods

### 2.1. Transfer of CVD-Grown Graphene

**Figure 1:**
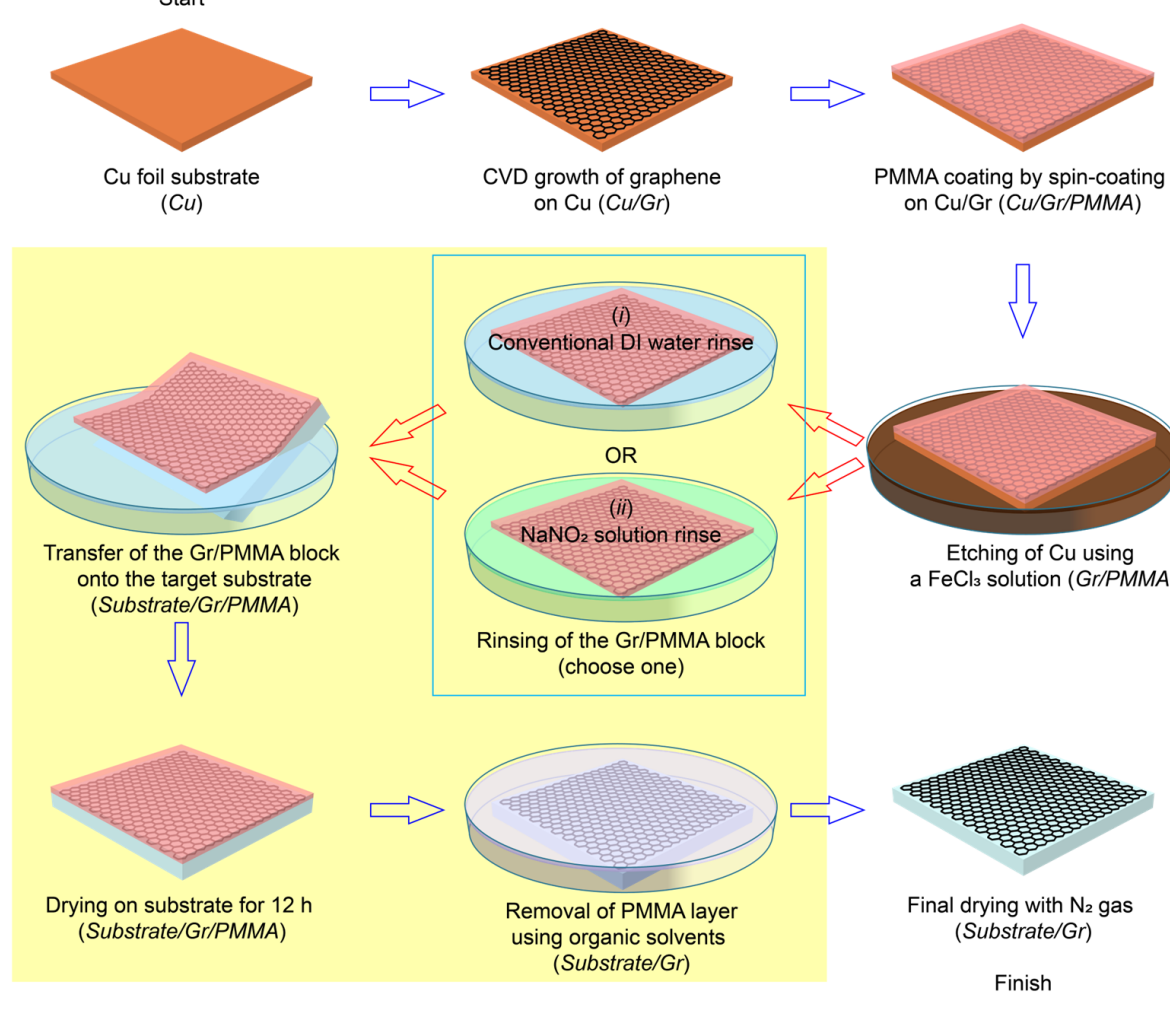
Schematic diagram of the graphene transfer process, illustrating two alternative rinsing methods: a conventional DI water rinse and a NaNO_2_ solution rinse. The process begins with CVD growth of graphene (Gr) on Cu, followed by spin coating of a PMMA layer (yielding Cu/Gr/PMMA) and subsequent Cu etching using an FeCl_3_ solution (producing Gr/PMMA). The rinsing step uses (blue box) either DI water or a NaNO_2_ solution before the Gr/PMMA block is transferred onto the target substrate. After the transfer, the PMMA layer is subsequently removed using organic solvents (final step highlighted in the yellow box), and the sample is dried with N_2_ gas, yielding the final clean graphene layer.

The procedure for transferring CVD-grown graphene onto arbitrary substrates, such as Si or glass, is as follows (Figure 1). In the figure, the Cu foil, graphene, and PMMA scaffold layer are distinctly labeled to highlight the NaNO_2_ solution rinse step compared to the conventional DI water rinse step. In the first step, graphene was synthesized on a clean Cu foil substrate via CVD. The Cu foil was placed inside a 2.5-inch quartz tube chamber and the temperature was elevated to 1000 °C under a flow of Ar gas (10 sccm). For graphene growth, a gas mixture of CH_4_ (30 sccm) and H_2_ (10 sccm) was introduced at a pressure of approximately 2.7 × 10^−2^ Pa [21,23,44].

In the second step, a PMMA solution (950PMMAC4, Mw: 495,000–950,000, 2–7 wt% in chlorobenzene, MicroChem, Kayaku Advanced Materials, Westborough, MA, USA) was spin-coated onto the graphene-covered Cu foil (Cu/Gr) at 3000 rpm for 60 s (EF-40P, E-FLEX, Gyeonggi, Republic of Korea) to form the PMMA support layer [45,46]. To remove graphene on the back side of the Cu foil, an atmospheric-pressure oxygen plasma (O_2_ flow: ~20 sccm, MyPL100, APP, Gyeonggi, Republic of Korea) was applied. The PMMA-coated Cu/Gr (Cu/Gr/PMMA) sample was cut into dimensions of approximately 4 mm × 20 mm and then floated on the surface of an aqueous FeCl_3_ solution (UN2582, FeCl_3_: 45 wt% in water, Transene Co. Inc., Denvers, MA, USA) at 50 °C for 20 min to completely etch the Cu foil.

After etching, the resulting Gr/PMMA block was thoroughly rinsed in either (i) conventional ultrapure DI water (conductivity ~5.6 × 10^−6^ S/m) for 10 min (Reference) or (ii) an aqueous NaNO_2_ solution (freshly prepared or stored) for 10 min (Sample or Comparative, respectively) prior to transfer onto the target substrate. For the NaNO_2_ solution (containing NO), the nominal concentration of NO (generated in situ) was ~2000 µM, optimized based on preliminary trials (see Section 2.2). The substrate with the transferred graphene was dried under reduced pressure (~1 Pa) for 1 h, followed by air drying for 12 h. To remove the PMMA support layer, the substrate was immersed sequentially in chloroform (1 h), monochlorobenzene (30 min), and chloroform again (30 min). This multi-step process ensured the effective removal of residual PMMA. The electrical characteristics of the transferred SLG were evaluated under ambient conditions using a Keithley 2400 source meter (Tektronix, Beaverton, OR, USA).

### 2.2. Treatment Methods

As shown in Figure 1, the NaNO_2_ solution rinse occurs immediately after Cu etching and prior to the final solvent steps, thereby removing ionic contaminants (FeCl_3_ by-products) and pre-conditioning the PMMA film for easy lift-off. Importantly, the NaNO_2_ solution rinse does not replace the role of organic solvents but rather complements it to achieve optimal cleaning. To assess how effectively the NaNO_2_ solution can neutralize ionic contaminants and loosen residual polymers on SLG, we devised and evaluated a straightforward rinsing protocol. The NaNO_2_ solution was prepared according to established protocols [47], where NaNO_2_ dissolved in DI water (2000 µM) then underwent a reaction process of NaNO_2_ + H^+^ → HNO_2_ + Na^+^ and 3HNO_2_ → H^+^ + 2NO + NO_3_^−^ + H_2_O at pH 3.5, resulting in a NO solution. In this study, Gr/PMMA blocks were immersed in a NaNO_2_ solution containing approximately 2000 μM NO immediately after Cu foil etching as an alternative to conventional DI water rinsing. The immersion process lasted 10 min. After the treatment, the Gr/PMMA block was transferred to the target substrate and subjected to drying under reduced pressure (~1 Pa) for 1 h, with this step followed by air drying for 12 h, as noted above. The PMMA support layer was then removed using the aforementioned sequential solvent process.

To evaluate the effectiveness of the surface treatment using an aqueous NaNO_2_ solution during the rinsing process of the transferred graphene, we employed three types of aqueous treatment media as rinsing materials: DI water (Reference), a freshly prepared aqueous NaNO_2_ solution stored for 0 h (Sample), and an aqueous NaNO_2_ solution stored for 24 h (Comparative).

### 2.3. Characterization

Electrochemical measurements of the prepared NaNO_2_ aqueous solutions were performed using a potentiostat/galvanostat (CS100E, Wuhan Corrtest Instruments Co., Ltd., Wuhan, China). The three-electrode cell used consisted of a platinum counter electrode (A115, Wuhan Corrtest Instruments Co., Ltd., Wuhan, China; diameter: 0.5 × 37 mm), a Ag/AgCl reference electrode (Sanxin, Shenyang, China) with a 3.5 M potassium chloride (KCl) electrolyte, and a bare glassy carbon (GC) working electrode (A104, Wuhan Corrtest Instruments Co., Ltd., Wuhan, China; diameter: 3 mm). Cyclic voltammetry (CV) measurements were taken over a potential range of 0.0 to +1.5 V at various scan rates ranging from 20 mV s^−1^ to 120 mV s^−1^. Each electrochemical experiment included at least two parallel measurements, and all measurements were carried out at room temperature. Additionally, the detection of NO_2_^−^ in the prepared NaNO_2_ aqueous solutions was investigated using a colorimeter (DR 900, HACH, Loveland, CO, USA).

To investigate the surface morphologies of the fabricated SLG layers, variations in their surface roughness levels were monitored using a non-contact 3D optical surface profiler (NV-2400, Nanosystem Co. Ltd., Daejeon, Republic of Korea) with a 100× objective lens with a lateral resolution of ~1 µm and a vertical resolution of ~0.5 µm. Additional conventional bright-field optical microscopy images (BA300 Pol., Motic Instruments, Richmond, VA, Canada) using 4× objective lens were also obtained to assess large-scale coverage and uniformity. The surface roughness and surface potential of the SLG specimens were characterized using non-contact AFM and simultaneous KPFM (FlexAFM, Nanosurf AG, Liestal, Switzerland). During the KPFM measurements, an AC voltage of 1 V at a frequency of 18 kHz was applied to a Pt/Ir-coated silicon tip (resonance frequency = 87 kHz, force constant = 3.9 N·m^−1^, NanoWorld Inc., Neuchâtel, Switzerland). Highly oriented pyrolytic graphite (HOPG, ZYB, Optigraph GmbH, Berlin, Germany) sample was used as a reference surface for work function calibration and tip integrity monitoring.

A surface composition analysis was performed using XPS (PHI 5000 VersaProbe, ULVAC-PHI Inc., Chigasaki, Japan) with a monochromated Al K-alpha source (E = 1486.6 eV). Raman spectroscopy was conducted on a Raman system (alpha300S with a 488 nm excitation laser, WITec, Ulm, Germany) using a 50× objective lens (NA = 0.7) and a 1200 grooves/mm diffraction grating. The laser power was ~3 mW, the acquisition time was 30 s, and 5 accumulations were averaged per spectrum. A silicon wafer reference at 520.7 cm^−1^ was used for calibration, with an accuracy of 1–2 cm^−1^. For each sample, representative Raman spectra were averaged from ten randomly selected points.

## 3. Results and Discussion

### 3.1. Characterization of the NaNO_2_ Aqueous Solution

**Figure 2:**
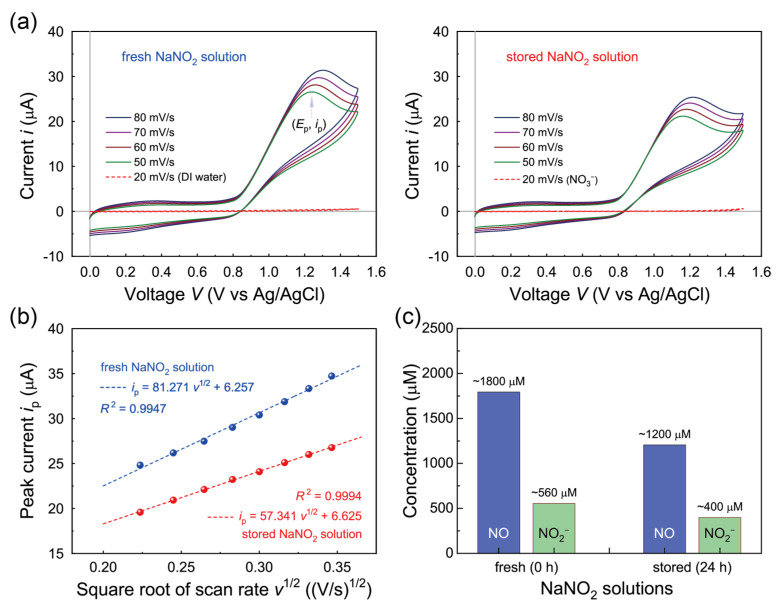
(**a**) Cyclic voltammograms of a fresh NaNO_2_ aqueous solution and DI water (red dotted curve) (**left**) and a NaNO_2_ aqueous solution stored for 24 h and a NO_3_^−^ solution (red dotted curve, 300 µM HNO_3_) (**right**) at different sweep rates (*v*, solid curves, pH ~3.2). The grey lines indicate the axes crossing at (0 V, 0 µA) of the CV curves. (**b**) Randles–Sevcik plots to estimate the concentrations of NO in the fresh and stored NaNO_2_ aqueous solutions. (**c**) Quantification of NO and NO_2_^−^ species in the NaNO_2_ aqueous solutions, derived from CV and colorimetric analyses.

To assess how effectively the aqueous NaNO_2_ solution neutralizes ionic contaminants (e.g., Cl^−^) and loosens residual PMMA on transferred SLG, we first characterized the properties of the NaNO_2_ solution used. CV measurements were employed to identify the reactive species in the NaNO_2_ aqueous solutions through their redox behaviors, using an unmodified bare GC working electrode without a supporting electrolyte. Cyclic voltammograms of a freshly prepared NaNO_2_ solution and a NaNO_2_ solution stored for 24 h, recorded at different scan rates (*v*), are shown in the left and right panels of Figure 2a, respectively. Measurements were performed with a platinum counter electrode and the bare GC working electrode, referenced to a Ag/AgCl electrode (vs. Ag/AgCl). A distinct irreversible oxidation peak was observed for both solutions in the potential range of +1.05 to +1.25 V vs. Ag/AgCl, indicative of electron transfer processes involving NO species at the GC electrode (NO → NO^+^ + 1e^−^) [48].

To quantify the reactive NO concentrations in both NaNO_2_ aqueous solutions, we analyzed the relationship between the oxidation peak current (*i*_p_) and the square root of the scan rate (*v*^1/2^) for the NO oxidation peaks (Figure 2b). The NO concentrations in the solutions were estimated using the modified Randles–Sevcik equation: *i*_p_ = 299 × 10^3^·*n*·*A*·*C*_0_·(*α*·*n_α_*·*D*)^1/2^·*v*^1/2^ + constant, where *A* represents the electrode area, *D* is the diffusion coefficient (2.54 × 10^−5^ cm^2^/s), and *C*_0_ is the NO concentration [49,50]. The calculated NO concentrations for the prepared NaNO_2_ aqueous solutions were approximately 1800 µM and 1200 µM for the fresh and stored NaNO_2_ solutions, respectively. Additionally, using the colorimeter, the concentrations of NO_2_^−^ in the prepared NaNO_2_ aqueous solutions were estimated to be approximately 560 µM and 400 µM for the fresh and stored solutions, respectively. These NaNO_2_ solutions, enriched with reactive NO species, were then used to neutralize ionic contaminants and loosen adsorbed polymer residues on SLG specimens transferred onto substrates.

### 3.2. Optical 3D Surface Morphologies of SLG Specimen Transferred to Glass Substrates

**Figure 3:**
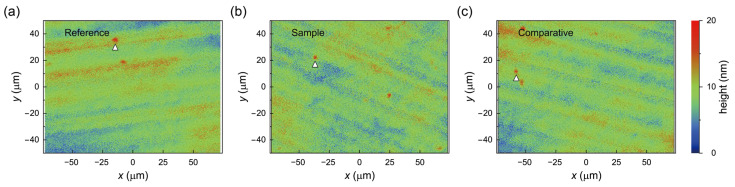
Three-dimensional optical microscopy surface images of SLG samples transferred onto glass substrates using different rinsing processes: (**a**) conventional DI water (Reference), (**b**) fresh NaNO_2_ aqueous solution (0 h, Sample), and (**c**) stored NaNO_2_ aqueous solution (24 h, Comparative). Arrows indicate the locations of residue spikes on the SLG samples.

Based on the CV observations, which demonstrated a strong interaction between NO and the carbon working electrode, we hypothesized that the high-concentration NO generated from the NaNO_2_ solution could affect the graphene surface. To evaluate the effectiveness of the aqueous NaNO_2_ rinsing process compared to conventional DI water rinsing during the SLG transfer process, we analyzed the surface morphologies of the treated SLG specimens by means of 3D optical microscopy (Figure 3). Figure 3a presents a representative 3D optical surface image of one of the SLG specimens transferred to a glass substrate rinsed with conventional DI water, the group of which is referred to as the Reference SLG samples. The surface of the Reference SLG appears relatively smooth, with a root mean square (RMS) height (*S*_q_) of approximately 2.0–2.5 nm, exhibiting only a few spikes (widths of approximately 4.9–7.3 µm and heights in the range of 9–12 nm) along with some microscale particles. These spikes and particles likely represent residual PMMA polymer on the SLG surface.

In contrast, the SLG specimens rinsed with a freshly prepared aqueous NaNO_2_ solution, referred to as the Sample SLG samples (Figure 3b), exhibit minor spikes (widths of approximately 4.5–5.9 µm and heights of ~7–11 nm) and relatively fewer microscale particles, with *S*_q_ values ranging from 1.8 to 2.1 nm. The observed decrease in the surface roughness, along with the fewer and smaller residual spikes or particles, suggests that the fresh NaNO_2_ solution rinse more effectively loosens and reduces PMMA residues on the SLG specimens compared to conventional DI water rinsing. This improved residue removal results in significantly smoother SLG surfaces, critical for optimizing the performance of graphene-based electronic devices.

**Figure A1:**
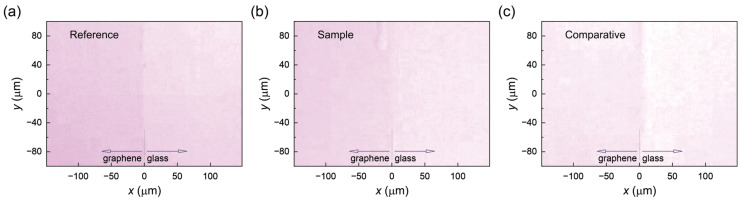
Bright-field optical microscopy images of SLG samples transferred onto glass substrates using different rinsing processes, namely (**a**) conventional DI water (Reference), (**b**) fresh NaNO_2_ aqueous solution (0 h, Sample), and (**c**) stored NaNO_2_ aqueous solution (24 h, Comparative), to illustrate the overall film uniformity and defect density.

Additionally, we evaluated SLG samples rinsed with an aqueous NaNO_2_ solution that had been stored for 24 h, referred to as the Comparative SLG samples (Figure 3c). The 3D optical surface image of the Comparative SLG similarly shows minor spikes (widths of approximately 3.6–5.9 µm, heights in the approximate range of 6–10 nm) and minor microscale particles, with *S*_q_ roughness values ranging from 1.9 to 2.2 nm, compared to the Reference SLG (Figure 3a). This indicates that the stored NaNO_2_ solution also reduces surface residues on the SLG specimens, although somewhat less effectively than the freshly prepared solution. Overall, our findings confirm that an aqueous NaNO_2_ solution rinse diminishes ionic and polymer contaminants more effectively than conventional DI water rinsing, thereby producing smoother SLG surfaces while also enhancing the surface quality. To confirm the large-area uniformity and coverage, conventional bright-field optical images are provided in Figure A1. Despite minor wrinkles, the coverage exceeds 95% across the samples, with no discernible macroscopic defects introduced by the NaNO_2_ solution rinse.

### 3.3. AFM Surface Morphologies and Surface Potentials of SLG Specimens Transferred to Glass Substrates

**Figure 4:**
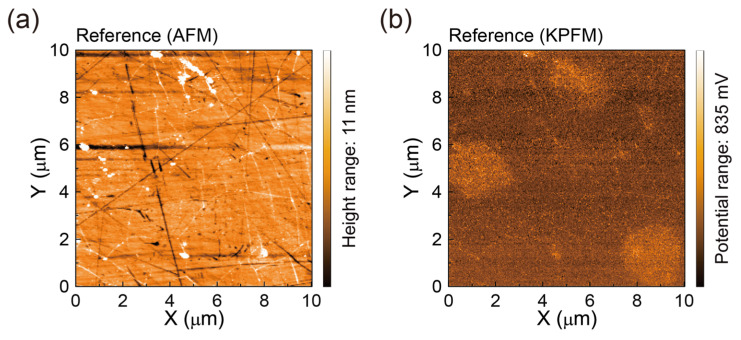
(**a**) AFM topographic image (10 µm × 10 µm) and (**b**) corresponding KPFM surface potential map of the Reference SLG transferred with PMMA polymer onto a glass substrate using conventional DI water rinsing.

Next, to evaluate the effects of the rinsing treatment, we investigated the sub-microscale surface morphologies and surface potentials [51] of the SLG specimens using non-contact AFM and simultaneous KPFM measurements (Figure 4). Figure 4a shows an AFM topographic image of the Reference SLG transferred to a glass substrate using the DI water rinsing treatment. The surface of the Reference SLG appears relatively smooth, with an RMS roughness value of approximately 1.03~1.55 nm, exhibiting only a few wrinkles and some nanoscale particles. The wrinkles are attributed to the difference in the thermal expansion coefficients between graphene and Cu [52], while the nanoscale particles (widths of ~0.33 ± 0.09 µm and heights of ~50.6 ± 19.7 nm) likely represent residual PMMA polymer on the SLG surface.

This AFM image is complemented by a corresponding local surface potential map [51] showing the surface contact potential differences (*V*_CPD_s) measured for the Reference SLG (Figure 4b). The surface potential map reveals relatively low *V*_CPD_s (−30 to −52 mV) in the flat areas of the SLG surface, in contrast to the relatively high surface potentials (*V*_CPD_s: 136–250 mV) near the submicron particle sites. The average *V*_CPD_ of the Reference SLG is estimated to be approximately 30.1 mV [52,53]. This low average *V*_CPD_ value is attributable to residues or adsorbate materials on the SLG surface, as the electrical properties of graphene are highly sensitive to substrate and environmental contamination [53] and strongly depend on the transfer conditions.

**Figure 5:**
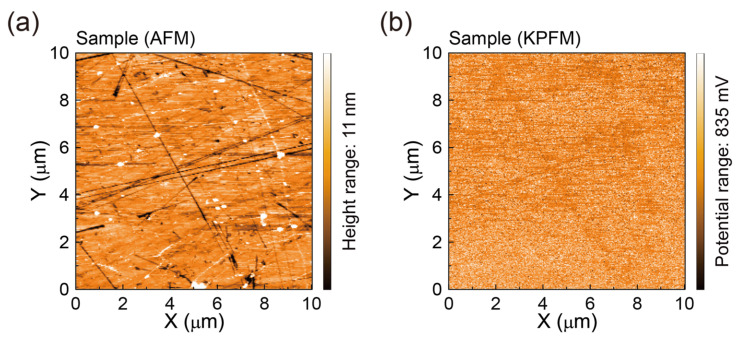
(**a**) AFM topographic image (10 µm × 10 µm) and (**b**) corresponding KPFM surface potential map of the Sample SLG transferred with PMMA polymer onto a glass substrate using the rinsing process with a fresh aqueous NaNO_2_ solution (Sample).

For comparison, we also analyzed the surface morphologies and surface potentials [51] of the Sample SLG rinsed with a freshly prepared aqueous NaNO_2_ solution (Figure 5). As shown in Figure 5a, the Sample SLG specimens exhibit AFM morphologies with an RMS roughness of 1.27–1.92 nm, comparable to that of the Reference SLG (Figure 4a). Notably, the AFM image of the Sample SLG reveals fewer submicron particles (widths of ~0.27 ± 0.07 µm and heights of ~36.2 ± 20.8 nm), some of which appear to be partially remaining residues, such as PMMA components on the SLG (Figure 5a). The reduced number of submicron particles is likely due to the effective removal of PMMA polymer residues from the SLG samples.

Moreover, the average surface potential *V*_CPD_ of the Sample SLG is significantly higher (~307 mV), indicating a substantial reduction in the amount of residue due to the fresh NaNO_2_ aqueous solution rinsing process (Figure 5b). The surface potential maps show relatively low *V*_CPD_s (201–226 mV) in the flat areas of the SLG surface, in contrast to the relatively high surface potentials (*V*_CPD_s: 364–400 mV) at the nanoscale particle sites. These results show that nanoscale PMMA residues elevate the surface potential of SLG, whereas the fresh NaNO_2_ solution rinse significantly reduces not only these polymeric residues but also adsorbed Cl^−^ from the FeCl_3_ etchant, thereby bringing the surface potential closer to intrinsic values [54]. These findings indicate that rinsing with a fresh NaNO_2_ solution more effectively diminishes both polymeric and ionic contaminants compared to conventional DI water rinsing, producing smoother SLG surfaces and improved electrical properties.

**Figure 6:**
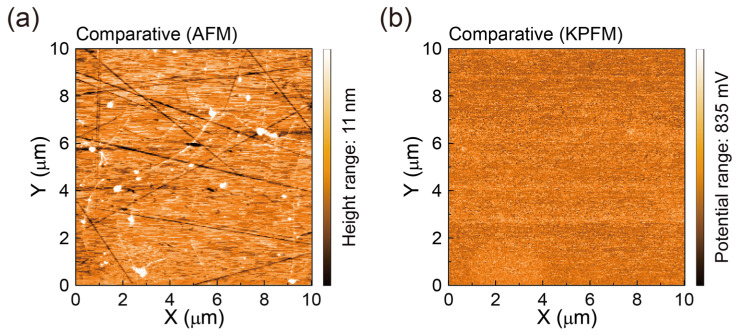
(**a**) AFM topographic image (10 µm × 10 µm) and (**b**) corresponding KPFM surface potential map of the Comparative SLG transferred with PMMA polymer onto a glass substrate using the rinsing process with a stored aqueous NaNO_2_ solution for 24 h (Comparative).

For further confirmation of the residue removal effect of the NaNO_2_ aqueous solution rinsing process, we investigated the surface morphologies and surface potentials [51] of a Comparative SLG specimen rinsed with a stored NaNO_2_ aqueous solution (Figure 6). As shown in Figure 6a, the Comparative SLG exhibits AFM morphologies with an RMS roughness of 1.42–1.55 nm, comparable to that of the Reference SLG (Figure 4a). Interestingly, the AFM image of the Comparative SLG shows fewer submicron particles (widths of ~0.32 ± 0.12 µm and heights of ~30.8 ± 18.4 nm), some of which appear to be partially residual, such as PMMA components on the SLG. This slight reduction in the number of submicron particles is likely due to the partial loosening and reduced adhesion of PMMA residues on the SLG surfaces.

In addition, the average surface potential *V*_CPD_ of the Comparative SLG is significantly higher (~205 mV), indicating a substantial reduction in the amount of residue due to the NaNO_2_ aqueous solution rinsing process (Figure 6b). The surface potential maps show comparatively low *V*_CPD_s (121–169 mV) in the flat areas of the SLG surface, in contrast to the relatively high surface potentials (*V*_CPD_s: 281–320 mV) at the nanoscale particle sites. While the overall RMS roughness appears only slightly reduced, KPFM measurements show a significant difference in the local contact potential, indicating that the NaNO_2_ treatment reduces doping-related shifts caused by adsorbed contaminants. The submicron residues that remain after DI water rinsing are clearly associated with higher (*p*-type doping) potentials compared to the relatively uniform surface after the NaNO_2_ solution rinsing process. These results confirm that NaNO_2_ solution rinsing more effectively diminishes both polymeric (PMMA) and ionic (Cl^−^) residues compared to conventional DI water rinsing, thereby producing smoother SLG surfaces with improved electrical properties. This is evidence that the NaNO_2_ solution rinse enhances the efficiency of the subsequent organic solvent washes, ensuring a more thoroughly cleaned SLG surface overall.

### 3.4. Work Functions and Compositions of SLG Specimens Transferred to Glass Substrates

**Figure 7:**
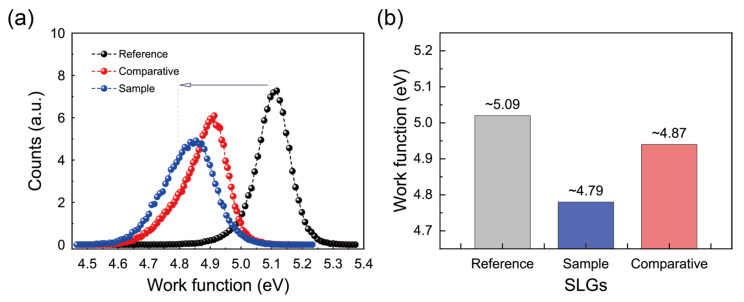
(**a**) *W*_Gr_ distributions derived from KPFM surface potential maps of SLG specimens transferred onto glass substrates using different rinsing treatments: conventional DI water rinse (Reference) and NaNO_2_ aqueous solution rinse (Sample and Comparative). The grey lines indicate the average values of the *W*_Gr_ distributions. (**b**) Schematic energy diagrams illustrating the electronic structures of the DI water-rinsed Reference SLG and the NaNO_2_ solution-rinsed Sample and Comparative SLG samples.

Next, we investigated the work functions of the SLG specimens (*W*_Gr_s) after the rinsing treatment by measuring the surface contact potential differences, i.e., the *V*_CPD_ values [51]. The *W*_Gr_ value was determined using the equation *W*_Gr_ = *W*_HOPG_ + [*V*_CPD_ (HOPG) − *V*_CPD_ (Gr)], where *W*_HOPG_ is the work function of HOPG (~4.6 eV) [51,55], and the *V*_CPD_ (Gr) values were measured using grounded SLG specimens [51,53]. From the KPFM maps (Figure 7a), we statistically derived the *W*_Gr_ distributions. As shown in Figure 7a, the estimated average *W*_Gr_ for the Reference SLG is approximately 5.09 eV, which is significantly higher than the intrinsic *W*_Gr_ range of clean SLG samples (4.5–4.8 eV) [51,53]. This elevated *W*_Gr_ is attributable to residues or adsorbate materials on the SLG surface, as discussed previously [56].

In contrast, the Sample SLG rinsed with a freshly prepared aqueous NaNO_2_ solution shows a lower *W*_Gr_ (~4.79 eV), indicating a significant decrease compared to the Reference SLG (Figure 7a). This reduced *W*_Gr_ is closer to the intrinsic range of the clean SLG (4.5–4.8 eV) [51,53], suggesting a rapid recovery of the intrinsic work function due to the substantial removal of surface residues. The decrease in *W*_Gr_ is likely due to the removal of not only PMMA polymer residues but also other contaminants, such as Cl^−^ ions from the FeCl_3_ etchant. These findings indicate that rinsing SLG with a fresh aqueous NaNO_2_ solution substantially decreases PMMA and Cl^−^ residues more effectively than a conventional DI water rinse.

For confirmation, we also examined the *W*_Gr_ values of Comparative SLG samples rinsed with the stored NaNO_2_ aqueous solution (Figure 7a). The Comparative SLG exhibited a slightly lower *W*_Gr_ of ~4.87 eV, indicating a small but noticeable decrease in *W*_Gr_ compared to the Reference SLG. This reduced *W*_Gr_ of the Comparative SLG is an intermediate value between those of the Reference and Sample SLG samples. This result confirms that rinsing SLG with an aqueous NaNO_2_ solution significantly reduces both PMMA and Cl^−^ contamination more effectively than a conventional DI water rinsing treatment. Without the NaNO_2_ solution rinsing step, certain deeply adsorbed ionic residues and tenacious polymer fragments persist—even after chloroform or monochlorobenzene rinses—resulting in a higher work function (~5.09 eV) and more pronounced doping. In contrast, samples subjected to the NaNO_2_ rinsing and organic solvent dissolving steps exhibit a work function closer to that of pristine graphene (~4.79 eV) as well as fewer polymeric clusters, highlighting the necessity of this combined approach. The *W*_Gr_s and associated electrical properties of the SLGs investigated in this study are summarized in Figure 7b and Table 1.

In addition, we measured the sheet resistance values (four-probe configuration) and extracted the conductivities and mobilities for each SLG sample (Table 1). As summarized in Table 1, the DI water-rinsed Reference SLG exhibits a sheet resistance of 653 Ω/□, conductivity of 4.55 × 10^6^ S/m, and mobility of 2.80 × 10^4^ cm^2^/V·s. In contrast, the fresh NaNO_2_-rinsed Sample SLG shows a higher sheet resistance of 883 Ω/□, lower conductivity (3.29 × 10^6^ S/m), and reduced mobility (2.03 × 10^4^ cm^2^/V·s), while the stored NaNO_2_-rinsed Comparative SLG presents intermediate values. These differences mainly arise from the impact of residual doping; the Reference SLG, despite its lower sheet resistance, exhibits a higher work function (5.09 eV), indicative of significant *p*-doping, likely due to residual PMMA and Cl^−^ impurities. In contrast, the fresh NaNO_2_ solution treatment, while increasing the sheet resistance and lowering the conductivity given the reduced carrier density, restores the work function closer to the intrinsic range (~4.79 eV), signifying the effective removal of doping contaminants. Residual dopants, such as Cl^−^ ions or polymer fragments, can enhance the carrier concentration, thereby lowering the sheet resistance, whereas they may also adversely affect the electronic properties required for reproducible high-performance devices. Thus, our results emphasize that achieving a de-doped, near-intrinsic graphene state is more desirable, even if it results in relatively higher sheet resistance. In addition, the standard deviations for the sheet resistance and work function measured across three independent CVD batches were typically within ±50 Ω/□ and ±0.05 eV, respectively, demonstrating consistent performance across multiple growth runs. Although minor variations in absolute electrical parameters are inevitable due to subtle differences in CVD growth conditions, the comparative improvement achieved by NaNO_2_ rinsing remains robust and reproducible.

### 3.5. C 1s XPS Spectra of SLG Specimens Transferred to Substrates

**Figure 8:**
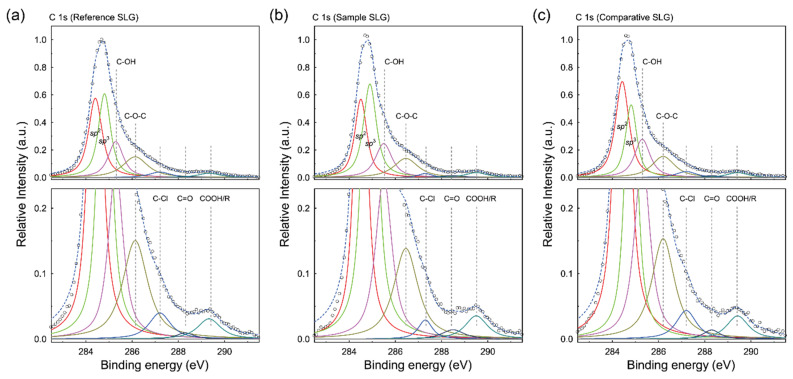
High-resolution XPS C 1s spectra (upper panels) and corresponding expanded views (lower panels) for Reference (**a**), Sample (**b**), and Comparative (**c**) SLG samples transferred onto ITO-coated glass substrates. The spectra were deconvoluted into seven components corresponding to distinct carbon bonding states: C=C (~284.4 eV, red curve), C–C (~284.8 eV, green curve), C–OH (~285.3 eV, magenta curve), C–O–C (~286.2 eV, dark yellow curve), C–Cl (centered at ~287.2 eV, blue curve), C=O (~288.3 eV, navy curve), and COOH/COOR (~289.4 eV, dark cyan curve).

To identify the residues on the SLG specimens, the surface compositions of these specimens on the substrates were analyzed by XPS. The XPS scan spectra (Figure 8) show strong photoelectron lines with binding energies around 285 eV, findings attributable to C 1s. For the SLG specimens studied here, the C 1s peaks were fitted to seven sub-peaks in the high-resolution spectra, as shown in the figure. The dominant peaks at approximately 284.4 eV and 284.8 eV are attributed to the intrinsic graphene structure (sp^2^ C=C and sp^3^ C–C bonds, respectively) [57,58]. In contrast, additional sub-peaks close to 285.3 eV, 286.2 eV, 288.3 eV, and 289.4 eV are primarily associated with various oxygen-containing groups from residual PMMA (C–OH, C–O–C, C=O, and COOH/COOR, respectively) [57,58]. Moreover, the sub-peak at approximately 287.2 eV is assigned to chloride-related species (C–Cl) [57,58], confirming the presence of adsorbed Cl^−^. Collectively, these features reveal that the C 1s spectrum reflects contributions from both pristine graphene and extraneous residues.

Notably, due to the rinsing treatment using the fresh NaNO_2_ solution instead of the typical treatment with DI water (Figure 8a,b), the C–O–C bond peak (286.2 eV) corresponding to the PMMA side chain decreased in terms of the XPS area percentage relative to the sp^2^ C=C bond, from 20% to 17%. These results indicate that the NaNO_2_ solution rinse leads to a significant reduction in the amount of PMMA residues on SLG during the subsequent organic solvent dissolving step. In particular, the fresh NaNO_2_ solution treatment resulted in a significant reduction in nanoscale polymer residues at structural defect sites on SLG, demonstrating that this cleaning effect is robust even in areas typically prone to greater residue adhesion.

In addition to the C-O-C bond peak, the XPS spectra revealed small but distinct peaks of C-Cl, likely to be residues from the FeCl_3_ etchant used in the Cu etching process. When Cl^−^ residues are adsorbed onto the SLG surface, the transfer of electrons from the SLG to Cl^−^ (chlorination) [54,59] induces unintentional *p*-doping of the SLG. For a detailed analysis, the relative XPS area percentages of the C-Cl bond peak (287.2 eV) to the C=C bond peak were estimated for the SLG specimens investigated here: the C-Cl bond peak clearly decreased in terms of the XPS area percentage relative to the C=C bond, from 4.5% to 2.1% (Figure 8a,b). These findings further demonstrate that the fresh NaNO_2_ solution effectively diminishes Cl^−^ contamination on SLG surfaces. Consequently, the NaNO_2_ solution rinse addresses both polymeric (PMMA) and ionic (Cl^−^) residues, leading to a more thorough surface cleaning than DI water alone.

For confirmation, we also examined the C-O-C and C-Cl bonding peaks in the C 1s XPS spectra of the Comparative SLG rinsed with the stored NaNO_2_ aqueous solution (Figure 8c). The Comparative SLG exhibited slight decreases in the area percentages of both the C-O-C bond peak (attributed to PMMA) and the C-Cl bond peak compared to the Reference SLG. Specifically, the C-O-C peak decreased from 20% to 18%, while the C-Cl peak slightly decreased from 4.5% to 4.4%. These reduced area percentages of the C-O-C and C-Cl bond peaks of the Comparative SLG are intermediate values between those of the Reference and Sample SLG specimens. These results confirm that rinsing SLG specimens with a NaNO_2_ aqueous solution substantially reduces both PMMA and Cl^−^ residues more effectively than the conventional DI water rinse.

Notably, comparing the C 1s XPS spectra of the Sample SLG (fresh solution) and Comparative SLG (stored solution) reveals that, despite similar NO_2_^−^ concentrations, the substantially higher NO level in the fresh NaNO_2_ solution produces a much greater reduction in polymeric and ionic residues on the SLG surface. This underscores the critical role of reactive NO species as the primary driver behind the improved cleaning effect. The process involves a synergistic combination of mild chemical oxidation and physical desorption: reactive NO partially oxidizes PMMA functional groups, weakening C–O–C bonds and increasing the hydrophilicity of the remaining fragments, thereby facilitating more efficient removal during the subsequent organic solvent step (chloroform/monochlorobenzene). In addition, NO species interact with Cl^−^ residues, forming chlorinated complexes or neutralizing their charge, which reduces electrostatic attraction onto the SLG surface and promotes desorption. Moreover, NO species induce Coulomb repulsive forces that aid in the physical detachment of negatively charged residue particles. Furthermore, NO-induced Cl^−^ desorption from the SLG surface not only removes ionic contaminants but also weakens PMMA adhesion by reducing the adsorbed Cl^−^ ions, which strongly interact with the dipolar side chains of PMMA polymers. This dual effect significantly enhances the overall efficiency of PMMA removal during the multi-step cleaning process. These combined chemical and electrostatic interactions enable NaNO_2_ solutions to reduce polymeric and ionic residues on SLG rapidly and substantially, i.e., within approximately 10 min, thus outperforming conventional DI water rinsing. Notably, extending the rinse beyond 10 min does not further enhance cleaning, indicating that most of the residue loosening and desorption occur within this timeframe.

Another potential pathway for the effective removal of PMMA residues from SLG via the subsequent process of organic solvent dissolving involves the disruption of strong hydrogen bonding interactions between the hydroxyl (-OH) groups on graphene and the carbonyl (C=O) groups in PMMA using aqueous NO. These hydrogen bonds enhance PMMA adhesion to SLG, complicating the complete removal of PMMA residues. NO can act as a selective chemical agent to break these interactions by reacting with hydroxyl groups and oxidizing them via the reaction C–OH + NO → C=O + HNO [60]. This oxidation eliminates the hydrogen donor (-OH), effectively weakening the (SLG)–OH⋯O=C (PMMA) hydrogen bond and reducing the PMMA adhesion strength. Consequently, the subsequent solvent dissolving step should more efficiently remove PMMA residues, leading to a cleaner graphene surface. This hypothesis is supported by the XPS analysis results, which show a relatively strong C=O sub-peak at 288.3 eV in the NO-treated Sample SLG compared to the Reference SLG, indicating a noticeable increase in the carbonyl content following the rinsing treatment with the NaNO_2_ solution (see Figure 8). It is therefore clear that this approach offers a simple yet effective modification to the graphene transfer process that in turn enhances the post-transfer surface purity while also minimizing polymer contamination.

### 3.6. Raman Spectra of SLG Specimens Transferred to Substrates

**Figure 9:**
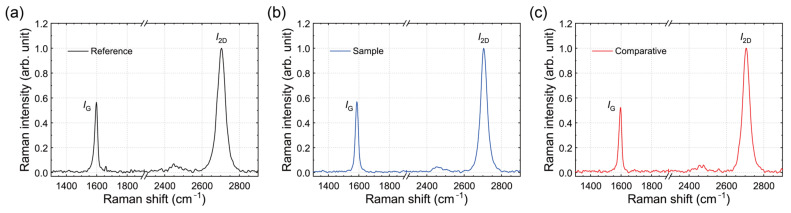
Raman spectra of the Reference (**a**), Sample (**b**), and Comparative (**c**) SLG specimens on SiO_2_/Si substrates.

To evaluate the cleaning efficacy of the NaNO_2_ aqueous solution rinsing treatment, we analyzed the Raman spectra of the SLG samples. The quality and doping level of graphene can be assessed from the positions and intensity ratios of the Raman peaks (Figure 9) [61]. The Raman spectra of the rinsed SLG specimens exhibited two prominent peaks: the G band (~1590 cm^−1^), corresponding to the E_2g_ vibrational mode of sp^2^-bonded carbon atoms, and the 2D band (~2655 cm^−1^), a second-order peak resulting from phonon scattering at the zone boundary [62]. Additionally, a weak-disorder-induced D band (~1330 cm^−1^) was observed, indicating minimal defects and sparse sp^3^ bond formation in these samples [63].

The integrated intensity ratios of the 2D band to the G band (*I*_2D_/*I*_G_) for all samples ranged from 3.59 to 4.12, confirming the high quality of the graphene studied here [7]. The G and 2D band positions for the Reference SLG were located at approximately 1595.5 ± 1.6 cm^−1^ and 2704.8 ± 2.9 cm^−1^, respectively. In contrast, the Sample SLG exhibited a clear downshift in the G and 2D bands to values of approximately 1590.9 ± 3.2 cm^−1^ and 2703.7 ± 1.6 cm^−1^, respectively. The Raman peak positions of the Comparative SLG (~1594.4 ± 2.9 cm^−1^ and ~2705.4 ± 1.3 cm^−1^) were nearly identical to those of the Reference SLG. By comparing our Raman results with previous studies correlating peak shifts with doping levels [7,64] and considering that practical factors such as sample heterogeneity and local substrate effects may introduce slight variations, we cautiously interpret the observed ~3–5 cm^−1^ downshifts in the Sample SLG as indicative of reduced *p*-doping. These Raman observations are further corroborated by KPFM-derived work function measurements, which show a restored value of approximately 4.79 eV, by XPS data revealing a lower Cl content, and by the sheet resistance values. Collectively, the combined data confirm that the NaNO_2_ solution rinsing treatment effectively reduces doping-inducing residue levels, thereby restoring the SLG to near-intrinsic electrical properties. Additionally, spot-based Raman checks of these submicron lumps reveal very weak, broad peaks indicative of polymer fragments or aggregates. Future work with detailed Raman mapping could further confirm the exact composition of each lump, but these preliminary checks support our assignment of PMMA-like contaminants.

In our study, the Reference SLG sample, rinsed with DI water, exhibited a work function of approximately 5.09 eV, which is significantly higher than the intrinsic range for pristine graphene (4.5–4.8 eV). This elevated work function is primarily attributed to residual doping, caused by Cl^−^ from the FeCl_3_ etchant and by remaining PMMA fragments that adhere to the graphene surface, particularly at defect sites or grain boundaries. These contaminants contribute to *p*-type doping by trapping charges and shifting the Fermi level away from the Dirac point. In contrast, the NaNO_2_ rinsing process effectively reduces these contaminants. Under mildly acidic conditions, NaNO_2_ generates reactive NO species that play a dual role: first, they chemically neutralize Cl^−^ ions (or Cl-based complexes), and second, they partially oxidize and loosen the residual PMMA. This chemical action facilitates a more efficient removal of contaminants during the subsequent organic solvent wash, thereby allowing the graphene’s Fermi level to relax toward the Dirac point. Consequently, the work function of the NaNO_2_-treated SLG is restored to approximately 4.79 eV, which is within the intrinsic range of pristine graphene. Complementary measurements further support this mechanism. For instance, KPFM data show a corresponding decrease in the local surface potential, consistent with reduced *p*-doping, while XPS analyses reveal lower intensity levels in Cl-related peaks. These combined observations confirm that the NaNO_2_-based rinsing method as proposed here not only cleans the graphene surface but also effectively “de-dopes” it, resulting in improved electrical properties. This approach demonstrates a practical, low-temperature, and reproducible means by which to mitigate unintentional doping, a critical factor for high-performance graphene-based devices.

In preliminary tests, a 10 min rinse at a NO concentration close to 2000 µM optimized the cleaning efficiency without damaging the graphene lattice. Extending the rinse beyond 10 min offered no further improvement, and significantly lower concentrations showed inferior cleaning results. Additionally, multiple CVD graphene batches subjected to the same protocol yielded reproducible outcomes (see also Table 1). It should be noted that unintended doping levels in conventionally prepared SLG specimens rinsed with DI water, caused by residues such as PMMA polymers and Cl^−^ ions, are neither precisely controllable nor reproducible. These uncertainties highlight the importance of the careful fabrication and evaluation of (opto)electronic devices incorporating SLG anodes to avoid misestimating the contributions of the SLG to the overall device performance. The NaNO_2_ solution rinsing process is highly recommended prior to integrating other physical or chemical doping methods into the fabrication of graphene-based (opto)electronic devices. This process provides a solid foundation for the production of controllable, reproducible, and high-performance devices by enabling precise *n*- or *p*-doping. Consequently, advanced electronic devices such as OLEDs, PV devices, and transistors can be developed without the complications associated with unintended *p*-doping caused by residual PMMA and Cl^−^ ions. Further details pertaining to the intentional doping effects of NaNO_2_ solution-rinsed SLG specimens on device performance capabilities will be reported in subsequent studies.

Future work will refine the NaNO_2_ rinsing approach further and explore its applicability to other 2D materials. In principle, this protocol can be extended to other 2D materials known to face challenges similar to polymer and ionic contamination during transfer processes, particularly when etching with scaffolds such as Cu, SiO_2_, or sapphire. For instance, the reactive aqueous NO generated in the rinsing solution could similarly remove doping ions or loosen residual polymer deposits on the surfaces of materials such as MoS_2_, WS_2_, or hBN. However, given that each 2D material exhibits unique stability and defect chemistry, further verification and optimization of certain parameters (e.g., rinsing time, solution pH) are necessary. Recent studies of doping control in other 2D semiconductors and hBN support the potential for developing tailored cleaning strategies that may ultimately improve device performance outcomes [65,66].

Finally, herein, we use NaNO_2_ to generate reactive NO species under mild acidic conditions that gently remove contaminants without any significant oxidation of graphene. While oxygen-based oxidants such as H_2_O_2_ and NaClO can also clean surfaces, they tend to be more aggressive and may introduce defects. Thus, our NO-based approach offers a favorable balance between cleaning efficiency and the preservation of the intrinsic properties of graphene. Further explorations of alternative oxidants remain of interest for future studies.

## 4. Conclusions

In summary, our study demonstrates that an aqueous NaNO_2_ rinse can significantly improve the quality of SLG by substantially reducing both residual PMMA and Cl^−^ contaminants. By harnessing reactive NO species under mildly acidic conditions, the NaNO_2_ solution rinse induces mild chemical oxidation and physical desorption, effectively diminishing these residues within approximately 10 min without compromising the intrinsic properties of the graphene. This treatment is essential for targeting ionic contaminants and weakening the adhesion of the PMMA layer, thereby enabling subsequent organic solvent (chloroform/monochlorobenzene) washes to remove the bulk polymer most efficiently. Together, this synergistic cleaning approach reduces unintentional doping and surface residues, restoring the graphene to a near-intrinsic state—an improvement that cannot be achieved by conventional DI water rinsing. In particular, AFM and XPS analyses confirmed that the SLG samples treated with NaNO_2_ exhibited cleaner and smoother surfaces than those rinsed with conventional DI water, with significantly reduced residue levels of PMMA polymer and Cl^−^ ions. Moreover, the treated SLG samples exhibited a restored work function of nearly 4.79 eV, close to the intrinsic work function of pristine graphene (~4.5–4.8 eV), representing a significant improvement over the DI water-rinsed SLG specimens (~5.09 eV). Furthermore, Raman spectroscopy confirmed that the NaNO_2_ treatment minimized unintentional *p*-doping effects, enabling controlled and reproducible doping levels critical for reliable device integration. Therefore, this transformative technique addresses long-standing challenges related to residue removal and unintended doping, ensuring consistent, high-quality SLG surfaces while maintaining compatibility with existing fabrication processes. Additionally, the NaNO_2_ rinsing process provides a simple, scalable, and time-efficient approach for cleaning SLG surfaces, facilitating the integration of this material into high-performance (opto)electronic devices, such as OLEDs, PV cells, and transistors, where precise doping control and surface quality are critical. Future studies will explore the possible effects of intentional doping and further optimization of the electronic properties of NaNO_2_-treated SLG, paving the way for specialized applications in advanced material systems and next-generation (opto)electronic technologies.

## Figures and Tables

**Table 1:** Summary of the characteristics of the SLG specimens studied here.

SLG Specimen	Rinsing with	Sheet Resistance (Ω/□)	Conductivity σ (×10^6^ S/m)	Mobility μ (×10^4^ cm^2^/Vs)	Work Function (eV)
Reference	DI water	653 ± 106	4.55 ± 0.84	2.80 ± 0.52	5.09 ± 0.05
Sample	Fresh NaNO_2_ solution	883 ± 49	3.29 ± 0.18	2.03 ± 0.11	4.79 ± 0.08
Comparative	Stored NaNO_2_ solution (24 h)	851 ± 102	3.45 ± 0.46	2.12 ± 0.28	4.87 ± 0.07

Measurements on three to five SLG samples from at least two independent CVD batches were used to derive mean values and standard deviations.

## Data Availability

The original contributions presented in this study are included in the article. Further inquiries can be directed to the corresponding author.
